# Taylor-Galerkin method for solving higher-order nonlinear complex differential equations

**DOI:** 10.1016/j.mex.2024.103078

**Published:** 2024-12-12

**Authors:** Md. Humayun Kabir, Md. Shafiqul Islam, Md. Kamrujjaman

**Affiliations:** a Department of Mathematics, Bangabandhu Sheikh Mujibur Rahman University, Kishoreganj 2300, Bangladesh; b Department of Applied Mathematics, University of Dhaka, Dhaka 1000, Bangladesh; c Department of Mathematics, University of Dhaka, Dhaka 1000, Bangladesh

**Keywords:** Finite Element Method, *MSC:* 92D25, 35K57 (primary), 35K61, 37N25, Complex differential equations, Taylor polynomials, Galerkin method, Residual error correction

## Abstract

The Galerkin approach for numerically resolving higher-order Complex Differential Equations (CDEs) in a rectangular domain in the complex plane is presented in this work. Taylor polynomial functions are used in this method as basis or weighted functions. The CDE is converted into a matrix equation by employing the proposed method. A system of linear and nonlinear equations with unknown Taylor coefficients for linear and nonlinear CDEs, respectively, is represented by the resultant matrix equation. Results pertaining to this method’s error analysis are discussed. The existing Taylor and Bessel Collocation methods are compared with the numerical results of the proposed method for linear CDEs, and the existing exact solutions and numerical results of the proposed method for nonlinear CDEs are also compared. The comparative results are displayed graphically for the real (ℜe) and imaginary (ℑm) parts, respectively, as well as in tabular form containing absolute error E(z) and maximum absolute error $L^{\infty }\;norm$. The methodology of this study focused on the Galerkin integral domain which is a rectangle shape in the complex plane and Taylor polynomial is the shape function. Matrix formulation procedure and iterative technique are implemented to find out the undetermined Taylor coefficients.

## Graphical abstract

**Figure:**
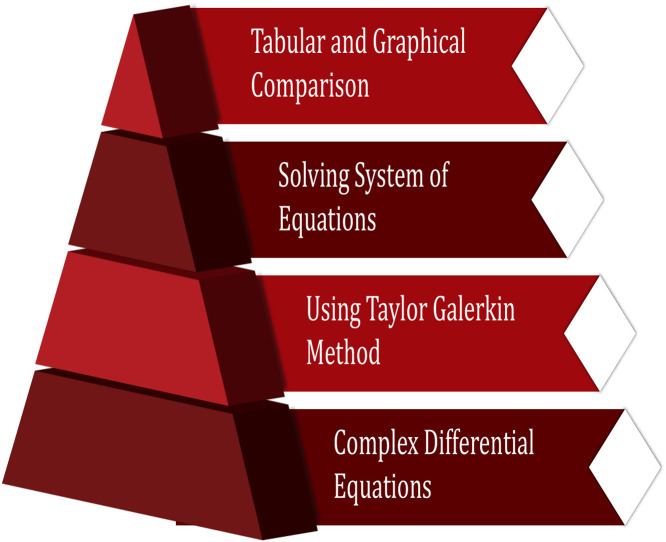

Specifications table

**Table:** 

Subject area:	Mathematics and Statistics
More specific subject area:	*Describe narrower subject area*
Name of your method:	*Finite Element Method*
Name and reference of original method:	Galerkin method using Taylor Polynomial
Resource availability:	*N.A.*

## Background

CDEs are prominent in quantum research and engineering. When a model based on mathematics is created to address a real-world physical phenomenon, it takes the shape of CDEs. By way of illustration, differential equations with a complex dependent variable are commonly used to explain the vibrations of a one-mass system with two degrees of freedom [1], [2]. In [2], [3], provides an overview of the various applications for complex dependent variables of differential equations. However, analytic techniques alone cannot provide a perfect solution. To overcome this challenge, numerical methods must be applied.

In the last few years, extensive research has been conducted on CDEs. Some of these studies include a geometric method in any domain that is based on meromorphic functions [4], a topological explanation of certain CDE solutions involving multi-valued coefficients [5], the complex oscillation of some linear CDE [6], the growth estimates of linear CDE [7], analytic functions in the complex plane: the polynomial and rational approximations [8], [9], the linear differential equations’ meromorphic solutions [10], [p,q]-order linear differential equations in the complex plane [11], a higher-order periodic linear differential equation problem [12], on complex domain the solution of IVP for retarded differential equations [13], an analytic method for the non-linear CDEs [14], the solution growth of algebraic systems of nonlinear CDEs [15], and also on meromorphic solutions and entire solutions by julia limiting directions and some function spaces solutions for the nonlinear CDEs have been studied in [16], [17], [18], [20], [21]. The following equation is a representation of the generalized $m^{\text{th}}$ order CDE with complex variable coefficients.

(1) $$\sum_{k=0}^{m}Q_{k}\left(z\right)f^{\left(k\right)}\left(z\right)=h\left(z\right);\;m\in N$$

where z is the complex variable, $Q_{k}\left(z\right)$ and h(z) are the analytic functions in the following rectangular domain D of the complex plane C

$$D=\left\{z\in C,z=x+iy,i=\sqrt{-1};a\leq x\leq b,c\leq y\leq d;\;a,b,c,d\in R\right\}.$$

Eq. (1) is a general CDE written down in derivative form given [6], [7], [8], [9], [19], [22], [23], with the following mixed conditions

(2) $$\sum_{k=0}^{m-1}\sum_{l=0}^{L}\left[b_{rk}f^{\left(k\right)}\left(\xi_{l}\right)+c_{rk}f^{\left(k\right)}\left(z_{0}\right)\right]=\lambda_{r};\;L\in N\;\text{and}\;r=0,1,2,\cdots ,\left(m-1\right).$$

where $b_{rk},\;c_{rk},$ and $\lambda_{r}$ are suitable real or complex constants;$\;\xi_{l},\;z_{0}\in D.$

Recently, solutions of CDEs have been estimated using numerous numerical techniques. For instance, Taylor Collocation approach for operational matrix method [22], [23], [24], Bessel polynomials method [25], Legendre polynomial method [26], [27], Euler polynomials method [28], orthogonal Bernstein polynomials method [29], Fibonacci polynomials method [30], Bernoulli polynomials method [31], and Hermite polynomials method [32], [33].

One of the most renowned weighted residual numerical methods for solving differential equations is the Galerkin method, which performs a significant role in the solution of differential equations numerically. For instance, the Galerkin method of Wavelet, Chebyshev, Taylor, Petrov, Legendre, Hermite, Bernstein, Exponential B-splines, and Bernoulli has been used to solve differential equations [34], [35], [36], [37], [38], [39], [40], integral and integro-differential equations [41], Volterra integro-differential equations [42], Fredholm integro-differential equations [43], eigenvalue problems [44], delay differential equations [45], [46], [47], Burger’s differential equations [48], KdV equations [49], nonlinear partial differential equations [50], [51], [52], and perturbed partial differential equations [53].

It has come to our attention that the previous research has not applied the Galerkin method for the numerical solution of CDEs. Since there is a research gap in this field, we presented a new technique for solving CDEs called the Taylor Galerkin Method (TGM). TGM uses Taylor series expansions for discretization, resulting in higher-order precision in temporal integration. This is especially useful for applications needing precise temporal resolution. TGM may successfully address nonlinear differential equations by adding Taylor expansions, which better represent nonlinear term behavior. While TGM can achieve high accuracy, it may incur additional computational costs due to the necessity for higher-order derivatives and the complexity of the numerical implementation. The collocation method is directly evaluating the governing equations at specific collocation points. The method can have lower computational overhead and cost for certain problems, especially when using fewer basis functions. However, this method can struggle with problems that involve sharp gradients or discontinuities, as the collocation points may not adequately capture local behavior. Poorly chosen points can lead to suboptimal convergence and inaccuracies. It can produce non-unique solutions, particularly when dealing with ill-posed problems or insufficiently defined basis functions. Both methods may encounter difficulties with boundary conditions, particularly for complicated geometries or where exact enforcement is required. However, TGM can be applied to a variety of spatial discretization techniques, including both structured and unstructured domains, increasing its application to a wide range of issues.

When dealing with complex shapes or domains, the TGM uses finite element discretization, which is highly flexible in terms of geometry. The domain (shape) is subdivided into smaller elements, and the method solves the problem over these elements using basis functions that are defined locally on each element. In this method, boundary conditions must be incorporated into both the time-stepping scheme (via the Taylor expansion) and the spatial discretization (Galerkin method).

**Fig. 1:**
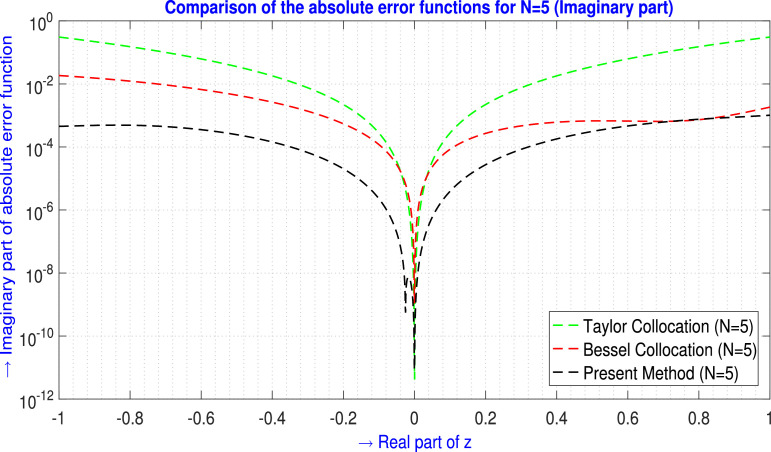
Visual depiction of the absolute error analysis for N=5 (ℑm part).

The immediate and a sample error analysis results are focused on Fig. 1. For a particular problem and the same number of basis functions, under those conditions TGM achieves higher-order accuracy by incorporating derivatives of the solution, which can significantly reduce error in the approximation, especially in smooth regions of the solution. On the other hand, since the Collocation method directly evaluates the governing equations at specific collocation points and the number of points is insufficient or poorly distributed, particularly near discontinuities or sharp gradients, it leads to larger errors.

The objective we set in the present research is to obtain an approximate solution ${\tilde{f}}_{N}\left(z\right)$ of equation (1) and subject to the condition Eq. (2), and want to express in terms of N degree Taylor polynomial in the following form; at the point $\;z=z_{0}$,

(3) $${\tilde{f}}_{N}\left(z\right)=\sum_{j=0}^{N}a_{j}\frac{{\left(z-z_{0}\right)}^{j}}{j!};\;z,z_{0}\in D\;\text{and}\;N\geq m.$$

where the unidentified Taylor coefficients $a_{j},\;j=0,1,2,\cdots ,N$ are to be determined. We will find out the unknown coefficients $a_{j}$ of the Eq. (3) by the proposed approach TGM. Since the proposed method is based on a Taylor series expansion, it may inherently handle higher-order derivatives more naturally than some other methods. This could lead to increased accuracy when approximating solutions that involve high spatial or temporal gradients. Computational complexity for the present method can grow rapidly, especially for multi-dimensional problems or when higher-order derivatives need to be accounted for. This could lead to a significant increase in memory allocation and high process time requirements, making TGM less efficient for very large-scale or real-time applications.

TGM needs to carefully handle boundary conditions (e.g., Dirichlet, Neumann, or mixed) for CDEs. Since the solution is complex-valued, both the real (ℜe) and imaginary (ℑm) parts must satisfy the boundary conditions.

## Method details: Phase 1

At first, we consider the approximate solution ${\tilde{f}}_{N}\left(z\right)$ and its kth derivative ${\tilde{f}}_{N}^{\left(k\right)}\left(z\right)$ in the following form [22], [24],

(4) $${\tilde{f}}_{N}\left(z\right)\equiv {\tilde{f}}_{N}^{\left(0\right)}\left(z\right)=\sum_{j=0}^{N}a_{j}\frac{{\left(z-z_{0}\right)}^{j}}{j!}=\sum_{j=0}^{N}a_{j}\theta_{j}\left(z\right)$$

and

(5) $${\tilde{f}}_{N}^{\left(k\right)}\left(z\right)\equiv \frac{d^{k}\left[{\tilde{f}}_{N}\left(z\right)\right]}{dz^{k}}=\frac{d^{k}}{dz^{k}}\left[\sum_{j=0}^{N}a_{j}\theta_{j}\left(z\right)\right]=\sum_{j=0}^{N}a_{j}\frac{d^{k}}{dz^{k}}\left[\theta_{j}\left(z\right)\right];\;k\in N.$$

where $\;\theta_{j}\left(z\right)=\frac{{\left(z-z_{0}\right)}^{j}}{j!}$ are considering the basis function for TGM.

We first gather every term in the CDE (1) on the left side to get the residual function [34], [35]. Now we substitute the relation (5) into the Eq. (1), then we obtain the corresponding residual function of ${\tilde{f}}_{N}\left(z\right)$ as follows,

(6) $$\begin{array}{cccc} R({\tilde{f}}_{N}) & =\sum_{k=0}^{m}Q_{k}\left(z\right){\tilde{f}}_{N}^{\left(k\right)}\left(z\right)-h\left(z\right) & & \\ \Rightarrow R({\tilde{f}}_{N}) & =\sum_{k=0}^{m}Q_{k}\left(z\right)\left[\sum_{j=0}^{N}a_{j}\frac{d^{k}}{dz^{k}}\left[\theta_{j}\left(z\right)\right]\right]-h\left(z\right). & \end{array}$$

We have to do to get a weighted residual is multiply the integral over the residual’s domain D by a weighting function w(z),

$$i.e.\int_{D}R\left({\tilde{f}}_{N}\right)w\left(z\right)dz.$$

By choosing (N+1) weight functions, $w_{i}\left(z\right)$ for i=0,1,2,⋯,N; and to find out the (N+1) unknown coefficients $a_{j}$ of Eq. (3), we have to solve (N+1) equations that result from putting these (N+1) weighted residuals to zero.

The (N+1) weighted residual for $w_{i}\left(z\right)$ is defined as follows

(7) $$R_{i}\left({\tilde{f}}_{N}\right)\equiv \int_{D}R\left(\tilde{f_{N}}\right)w_{i}\left(z\right)dz\;\;\text{for}\;i=0,1,\cdots ,N.$$

Since the weighted residual method requires [36], [39], [40], [45]

$$R_{i}\left({\tilde{f}}_{N}\right)=0\;\;\text{for}\;i=0,1,\cdots ,N.$$

That implies

(8) $$\;\int_{D}R\left(\tilde{f_{N}}\right)w_{i}\left(z\right)dz=0\;\;\text{for}\;i=0,1,\cdots ,N.$$

The main task of the TGM is to match the weight functions to the basis functions of the approximate solution ${\tilde{f}}_{N}\left(z\right)$. That is,

(9) $$w_{i}\left(z\right)=\theta_{i}\left(z\right)\;\;\text{for}\;i=0,1,\cdots ,N.$$

Now, by substituting the Eq. (6) and (9) into the Eq. (8), then the Galerkin weighted residual equations or simply the Galerkin equations are [37], [43]

(10) $$\begin{array}{cccc} & \int_{\;D}\left[\left(\sum_{k=0}^{m}Q_{k}\left(z\right)\left(\sum_{j=0}^{N}a_{j}\frac{d^{k}}{dz^{k}}\left[\theta_{j}\left(z\right)\right]\right)-h\left(z\right)\right)\theta_{i}\left(z\right)\right]dz=0 & & \\ & \Rightarrow \int_{\;D}\left[\sum_{k=0}^{m}Q_{k}\left(z\right)\left(\sum_{j=0}^{N}a_{j}\frac{d^{k}}{dz^{k}}\left[\theta_{j}\left(z\right)\right]\right)\theta_{i}\left(z\right)\right]dz=\int_{\;D}h\left(z\right)\theta_{i}\left(z\right)dz & & \\ & \Rightarrow \sum_{j=0}^{N}a_{j}\left[\int_{\;D}\left(\sum_{k=0}^{m}Q_{k}\left(z\right)\left(\frac{d^{k}}{dz^{k}}\left[\theta_{j}\left(z\right)\right]\right)\theta_{i}\left(z\right)\right)dz\right]=\int_{\;D}h\left(z\right)\theta_{i}\left(z\right)dz\;\;\text{for}\;i=0,1,\cdots ,N. & \end{array}$$

The Eq. (10) can be written in conventional matrix form as follows,

(11) [K]{A}={C}

where

(12) $$K=\left[k_{i,j}\right];\;k_{i,j}=\int_{\;D}\left[\sum_{k=0}^{m}Q_{k}\left(z\right)\left(\frac{d^{k}}{dz^{k}}\left[\theta_{j}\left(z\right)\right]\right)\theta_{i}\left(z\right)\right]dz\;\;\text{for}\;i,j=0,1,\cdots ,N$$

and

(13) $$C={\left[c_{i}\right]}^{T};\;c_{i}=\int_{\;D}h\left(z\right)\theta_{i}\left(z\right)dz\;\;\text{for}\;i=0,1,\cdots ,N$$

and

(14) $$A={\left[a_{j}\right]}^{T}\;\;\;\text{for}\;j=0,1,2,\cdots ,N.$$

The appropriate matrix configuration for the mixed conditions (2) can be obtained in the following manner [23], [25],

(15) $$\begin{array}{cccc} & \sum_{k=0}^{m-1}\sum_{l=0}^{L}\left[b_{rk}\frac{d^{k}}{dz^{k}}\left(\sum_{j=0}^{N}a_{j}\theta_{j}\left(\xi_{l}\right)\right)+c_{rk}\frac{d^{k}}{dz^{k}}\left(\sum_{j=0}^{N}a_{j}\theta_{j}\left(z_{0}\right)\right)\right]=\lambda_{r} & & \\ & \Rightarrow \sum_{j=0}^{N}a_{j}\left[\sum_{k=0}^{m-1}\sum_{l=0}^{L}\left(b_{rk}\frac{d^{k}\theta_{j}\left(\xi_{l}\right)}{dz^{k}}+c_{rk}\frac{d^{k}\theta_{j}\left(z_{0}\right)}{dz^{k}}\right)\right]=\lambda_{r}\;\;\text{for}\;r=0,1,2,\cdots ,\left(m-1\right). & \end{array}$$

The Eq. (15) can be written in conventional matrix form as follows,

(16) $$\left[U\right]\left\{A\right\}=\left\{\lambda_{r}\right\}$$

where

(17) $$U=\left[u_{r,j}\right];\;u_{r,j}=\sum_{k=0}^{m-1}\sum_{l=0}^{L}\left(b_{rk}\frac{d^{k}}{dz^{k}}\theta_{j}\left(\xi_{l}\right)+c_{rk}\frac{d^{k}}{dz^{k}}\theta_{j}\left(z_{0}\right)\right)\;\;\text{for}\;r=0,1,\cdots ,\left(m-1\right);\;j=0,1,\cdots ,N.$$

## Method details: Phase 2

The augmented matrix form of Eq. (11) becomes [22], [24]

(18) $$\begin{array}{cccc} \left[K:C\right] & =[k_{i,j}:c_{i}]\;;\;i,j=0,1,2,\cdots ,N & & \\ \Rightarrow [K:C] & =\left[\begin{array}{cccccc} k_{0,0} & k_{0,1} & \cdots & k_{0,N} & : & c_{0}\\ k_{1,0} & k_{1,1} & \cdots & k_{1,N} & : & c_{1}\\ \vdots & \vdots & \vdots & \vdots & \vdots & \vdots \\ \vdots & \vdots & \vdots & \vdots & \vdots & \vdots \\ k_{N-1,0} & k_{N-1,1} & \cdots & k_{N-1,N} & : & c_{N-1}\\ k_{N,0} & k_{N,1} & \cdots & k_{N,N} & : & c_{N} \end{array}\right] & \end{array}$$

which contains (N+1) rows.

The augmented matrix form of Eq. (16) yields

(19) $$\begin{array}{cccc} \left[U:\lambda \right] & =\left[u_{r,j}:\lambda_{r}\right]\;;\;r=0,1,2,\cdots ,\left(m-1\right);\;j=0,1,\cdots ,N & & \\ \Rightarrow [U:\lambda ] & =\left[\begin{array}{cccccc} u_{0,0} & u_{0,1} & \cdots & u_{0,N} & : & \lambda_{0}\\ u_{1,0} & u_{1,1} & \cdots & u_{1,N} & : & \lambda_{1}\\ \vdots & \vdots & \vdots & \vdots & \vdots & \vdots \\ u_{m-1,0} & u_{m-1,1} & \cdots & u_{m-1,N} & : & \lambda_{m-1} \end{array}\right] & \end{array}$$

which contains m rows.

Thus, it is possible to determine the unidentified Taylor coefficients $a_{j};\;j=0,1,2,\cdots ,N$, associated with the equivalent solution of the problem (1), which is composed of Eq. (11) and conditions (16), by swapping the m row matrices (19) out the last m rows of the augmented matrix (18). We have the new augmented matrix form as follows [23], [25],

(20) $$\left[K^{*}:C^{*}\right]=\left[\begin{array}{cccccc} k_{0,0} & k_{0,1} & \cdots & k_{0,N} & : & c_{0}\\ k_{1,0} & k_{1,1} & \cdots & k_{1,N} & : & c_{1}\\ \vdots & \vdots & \vdots & \vdots & \vdots & \vdots \\ k_{N-m,0} & k_{N-m,1} & \cdots & k_{N-m,N} & : & c_{N-m}\\ u_{0,0} & u_{0,1} & \cdots & u_{0,N} & : & \lambda_{0}\\ \vdots & \vdots & \vdots & \vdots & \vdots & \vdots \\ u_{m-1,0} & u_{m-1,1} & \cdots & u_{m-1,N} & : & \lambda_{m-1} \end{array}\right]$$

or, the equivalent matrix equation

(21) $$\left[K^{*}\right]\left\{A\right\}=\left\{C^{*}\right\}.$$

If $\det K^{*}\neq 0,$ we can rewrite the Eq. (21) as

(22) $$\left\{A\right\}={\left[K^{*}\right]}^{-1}\left\{C^{*}\right\}$$

and it is unique to determining the column matrix A which is the unknown coefficient of the Taylor polynomial (3).

Therefore, there exists a unique solution to the $m^{\text{th}}$ order linear CDE with variable coefficients under the given conditions. For nonlinear CDE, we construct a nonlinear system of equations with undetermined Taylor coefficients. We can solve this nonlinear system of equations numerically by well-known iterative techniques such as Newton’s Method, Levenberg-Marquardt, and Broyden’s Method [54], [55]. For a better approximation, we have to increase the degree of N polynomial (3).

## Method validation: based on error analysis

In this section, by using the residual function of the $m^{\text{th}}$ order CDE provided by Eq. (1), we estimate the error for the proposed method. Next, we demonstrate how to use this estimation to improve the approximate solution of the equation, known as the corrected solution. Finally, the Taylor theorem is utilized to determine an error bound on the corrected solution’s error [45], [46], [47]. Let us consider the residual function of Eq. (1) as follows:

(23) $$R\left(z\right)=\sum_{k=0}^{m}Q_{k}\left(z\right)f^{\left(k\right)}\left(z\right)-h\left(z\right)=0.$$

Now substitute the approximate solution ${\tilde{f}}_{N}\left(z\right)$ in place of f(z) to the Eq. (23), we get

(24) $$R_{N}\left(z\right)=\sum_{k=0}^{m}Q_{k}\left(z\right){\tilde{f}}_{N}^{\left(k\right)}\left(z\right)-h\left(z\right)$$

as the residual function of ${\tilde{f}}_{N}\left(z\right)$. Subtracting Eq. (23) from Eq. (24), we obtain

(25) $$\sum_{k=0}^{m}Q_{k}\left(z\right)E_{N}^{\left(k\right)}\left(z\right)=-R_{N}\left(z\right)$$

which is just as Eq. (1) with non-homogeneous term $-R_{N}\left(z\right)$ instead of h(z) and $f\left(z\right)-\tilde{f_{N}}\left(z\right)$ is restored by $E_{N}\left(z\right)$. Since the approximate solution ${\tilde{f}}_{N}\left(z\right)$ also assure the mixed condition (2), we obtain the corresponding homogeneous condition

(26) $$\sum_{k=0}^{m-1}\sum_{l=0}^{L}\left[b_{rk}E_{N}^{\left(k\right)}\left(\xi_{l}\right)+c_{rk}E_{N}^{\left(k\right)}\left(z_{0}\right)\right]=0;\;r=0,1,2,\cdots ,\left(m-1\right).$$

This is the mixed condition of the Eq. (25). Now using the solution method described in Method details: Phase 1 and Method details: Phase 2 section to obtain an approximation solution $E_{N,M}\left(z\right)$ to Eq. (25), where M is any positive integer. Lastly, we apply this approximation to obtain the approximate corrected solution for Eq. (1), which is given by

(27) $${\tilde{f}}_{N,M}\left(z\right)={\tilde{f}}_{N}\left(z\right)+E_{N,M}\left(z\right)$$

where the actual error of ${\tilde{f}}_{N,M}\left(z\right)$ is given by $f\left(z\right)-{\tilde{f}}_{N,M}\left(z\right)$.

In the following theorem, the truncation error of the Taylor expansion for the exact solution of Eq. (1) is used to evaluate the error bound for the approximate solution ${\tilde{f}}_{N}\left(z\right)$.

Theorem 1 *Let*${\tilde{f}}_{N}\left(z\right)$*be the approximate solution and*f(z)*be the exact solutions of*Eq. (1)*. If*f(z)*has*(N+1)*times continuous derivative, then the error bound for the absolute error is given by*

$$\left|f\left(z\right)-{\tilde{f}}_{N}\left(z\right)\right|\leq \left|R_{N}^{T}\left(z\right)\right|+\left|f_{N}^{T}\left(z\right)-{\tilde{f}}_{N}\left(z\right)\right|.$$

*Where*$f_{N}^{T}\left(z\right)$*denotes the*Nth*degree Taylor polynomial of*f(z)*around the point*$z=z_{0}\in D$*and*$R_{N}^{T}\left(z\right)$*represents its Cauchy form remainder term**[46]**.*

Proof The Taylor series can be rewritten with reminder term of f(z) around the point $z_{0}\in D$ as

$$f\left(z\right)=\sum_{j=0}^{N}\frac{{\left(z-z_{0}\right)}^{j}}{j!}f^{\left(j\right)}\left(z_{0}\right)+R_{N}^{T}\left(z\right).$$

Where

$$R_{N}^{T}\left(z\right)=\frac{1}{2\pi i}\oint_{\gamma }{\left(\frac{z-z_{0}}{t-z_{0}}\right)}^{\left(N+1\right)}\frac{f(t)}{t-z}dt$$

is the Cauchy form reminder term of the Taylor expansion of f(z) and this contour integral is evaluated around the circle γ which centered at $z_{0}$, such that γ⊂D [19]. Consequently, $R_{N}^{T}\left(z\right)=f\left(z\right)-f_{N}^{T}\left(z\right)$. By using this in conjunction with the triangle inequality, we get

$$\begin{array}{cc} \left|f\left(z\right)-{\tilde{f}}_{N}\left(z\right)\right| & =\left|f\left(z\right)-{\tilde{f}}_{N}\left(z\right)+f_{N}^{T}\left(z\right)-f_{N}^{T}\left(z\right)\right|\\ & \leq \left|f\left(z\right)-f_{N}^{T}\left(z\right)\right|+\left|f_{N}^{T}\left(z\right)-{\tilde{f}}_{N}\left(z\right)\right|\\ & =\left|R_{N}^{T}\left(z\right)\right|+\left|f_{N}^{T}\left(z\right)-{\tilde{f}}_{N}\left(x\right)\right|. \end{array}$$

As a result, we have located an upper bound for the absolute error based on the Taylor truncation error of the exact solution. □

## Method validation: based on numerical data

This section will demonstrate the numerical solution of three linear and two nonlinear CDEs by applying the proposed method. Nonlinear CDEs often involve terms like products of the unknown solution or its derivatives, which complicate both the iterative solution and error correction. The Newton-Raphson or Picard iteration methods are used to decouple these nonlinear terms at each iteration. All results are presented numerically, along with the exact solution and comparison. Since the N degree polynomial (3) is an approximate solution of Eq. (1), when the approximation solutions ${\tilde{f}}_{N}\left(z\right)$ and exact solution f(z) are substituted in the following equation, we can evaluate the absolute errors $E_{N}\left(z\right)$ at the subsequent particular points within the specified domain; that is, for $z=z_{j}\;\in \;D,$

(28) $$E_{N}\left(z_{j}\right)=\left|\tilde{f_{N}}\left(z_{j}\right)-f\left(z_{j}\right)\right|.$$

The absolute error $E_{N}\left(z\right)$ diminishes when N grows to a significant size.

We can also evaluate the maximum absolute error $L^{\infty }\;norm$ as follows:

(29) $$L^{\infty }\;norm=\max\left[E_{N}\left(z_{j}\right)\right].$$

Example 1 Let us examine the second-order non-homogeneous CDE that is linear and has variable coefficients [24], [25],

(30) $$f^{{}^{\prime \prime }}\left(z\right)+zf\left(z\right)=e^{z}+ze^{z};\;z\in C.$$

Where $m=2,\;Q_{0}\left(z\right)=z,\;Q_{1}\left(z\right)=0,\;Q_{2}\left(z\right)=1,\;h\left(z\right)=e^{z}+ze^{z}$ and subject to the initial conditions are

(31) $$f\left(0\right)=1,\;f^{\prime }\left(0\right)=1.$$

The corresponding transcendental entire solution of Eq. (30) is $f\left(z\right)=e^{z}$ and now consider an approximate solution ${\tilde{f}}_{5}\left(z\right)$ by the N=5 degree Taylor polynomial at $z_{0}=0$ in the following form

(32) $${\tilde{f}}_{5}\left(z\right)=\sum_{j=0}^{5}a_{j}\frac{z^{j}}{j!}.$$

Thus, we have $\;\theta_{j}\left(z\right)=\frac{z^{j}}{j!}\;\text{for}\;j=0,1,\cdots ,5$ and $\;\theta_{i}\left(z\right)=\frac{z^{i}}{i!}\;\text{for}\;i=0,1,\cdots ,5$

Assume the Galerkin integral domain $D=\left\{z\;\in \;C,\;z=x+iy,i=\sqrt{-1};\;-1\leq x\leq 1,\;-1\leq y\leq 1\right\}$. From Eq. (18), we obtain the augmented matrix by using Eq. (12) and (13) as follows:

$\left[K:C\right]=\left[\begin{array}{cccccccc} 0 & \frac{-4}{3}+\frac{4i}{3} & 2+2i & \frac{-4}{15}-\frac{4i}{15} & \frac{-2}{3}+\frac{2i}{3} & \frac{2}{105}-\frac{2i}{105} & : & -0.3103+3.6453i\\ \frac{-4}{3}+\frac{4i}{3} & 0 & \frac{-4}{5}-\frac{4i}{5} & \frac{-4}{3}+\frac{4i}{3} & \frac{2}{21}-\frac{2i}{21} & \frac{-4}{15}-\frac{4i}{15} & : & -3.6136+1.4915i\;\\ 0 & \frac{-4}{5}-\frac{4i}{5} & \frac{-2}{3}+\frac{2i}{3} & \frac{4}{21}-\frac{4i}{21} & \frac{-2}{5}-\frac{2i}{5} & \frac{2}{135}+\frac{2i}{135} & : & -1.6120-0.7535i\;\\ \frac{-4}{15}-\frac{4i}{15} & 0 & \frac{4}{21}-\frac{4i}{21} & \frac{-4}{15}-\frac{4i}{15} & \frac{2}{81}+\frac{2i}{81} & \frac{4}{63}-\frac{4i}{63} & : & -0.2513-0.7562i\;\\ 0 & \frac{2}{21}-\frac{2i}{21} & \frac{-1}{15}-\frac{i}{15} & \frac{2}{81}+\frac{2i}{81} & \frac{1}{21}-\frac{i}{21} & \frac{-1}{495}+\frac{i}{495} & : & 0.1046-0.1764i\;\\ \frac{2}{105}-\frac{2i}{105} & 0 & \frac{2}{235}+\frac{2i}{235} & \frac{2}{105}-\frac{2i}{105} & \frac{-1}{495}+\frac{i}{495} & \frac{2}{405}+\frac{2i}{405} & : & 0.0553-0.0161i \end{array}\right]$.

From Eq. (19), the augmented matrix form for the initial condition (31) is

$$\left[U:\lambda \right]=\left[\begin{array}{cccccccc} 1 & 0 & 0 & 0 & 0 & 0 & : & 1\\ 0 & 1 & 0 & 0 & 0 & 0 & : & 1 \end{array}\right].$$

From Eq. (20), we obtain the new augmented matrix form by applying the initial condition as follows:

$$\left[K^{*}:C^{*}\right]=\left[\begin{array}{cccccccc} 0 & \frac{-4}{3}+\frac{4i}{3} & 2+2i & \frac{-4}{15}-\frac{4i}{15} & \frac{-2}{3}+\frac{2i}{3} & \frac{2}{105}-\frac{2i}{105} & : & -0.3103+3.6453i\\ \frac{-4}{3}+\frac{4i}{3} & 0 & \frac{-4}{5}-\frac{4i}{5} & \frac{-4}{3}+\frac{4i}{3} & \frac{2}{21}-\frac{2i}{21} & \frac{-4}{15}-\frac{4i}{15} & : & -3.6136+1.4915i\;\\ 0 & \frac{-4}{5}-\frac{4i}{5} & \frac{-2}{3}+\frac{2i}{3} & \frac{4}{21}-\frac{4i}{21} & \frac{-2}{5}-\frac{2i}{5} & \frac{2}{135}+\frac{2i}{135} & : & -1.6120-0.7535i\;\\ \frac{-4}{15}-\frac{4i}{15} & 0 & \frac{4}{21}-\frac{4i}{21} & \frac{-4}{15}-\frac{4i}{15} & \frac{2}{81}+\frac{2i}{81} & \frac{4}{63}-\frac{4i}{63} & : & -0.2513-0.7562i\;\\ 1 & 0 & 0 & 0 & 0 & 0 & : & 1\;\\ 0 & 1 & 0 & 0 & 0 & 0 & : & 1 \end{array}\right]$$.

Here, $\det(K^{*})\neq 0$ and so by solving the linear system of equations $\left[K^{*}\right]\left\{A\right\}=\left\{C^{*}\right\}$, the unknown Taylor coefficients $a_{j}$ become

$$\left[\begin{array}{c} a_{0}\\ a_{1}\\ a_{2}\\ a_{3}\\ a_{4}\\ a_{5} \end{array}\right]=\left[\begin{array}{c} 1.000000000000000+0.000000000000000i\\ 1.000000000000000+0.000000000000000i\\ 1.014201842129353+0.001179180289414i\\ 1.008159146912473-0.001763403006139i\\ 0.990894060694918+0.141332871646214i\\ 0.995534591695822+0.111337779580632i \end{array}\right].$$

Therefore, the approximate solution (32) of Eq. (30) is

$\tilde{f_{5}}\left(z\right)=1+z+\left(1.014201842129353+0.001179180289414i\right)\frac{z^{2}}{2!}+\left(1.008159146912473-0.001763403006139i\right)\frac{z^{3}}{3!}\;\;+\left(0.990894060694918+0.141332871646214i\right)\frac{z^{4}}{4!}+\left(0.995534591695822+0.111337779580632i\right)\frac{z^{5}}{5!}.$

Similarly, we can also calculate the approximate solution of Eq. (30) for N=9. That is

$\tilde{f_{9}}\left(z\right)=1.0+z+\left(0.999997844737495-0.000000101718649i\right)\frac{z^{2}}{2!}+\left(0.999998850562165+0.000000108052043i\right)\frac{z^{3}}{3!}\;\;+\left(1.000003432575726-0.000077559255736i\right)\frac{z^{4}}{4!}+\left(1.000000124862018-0.000050328900171i\right)\frac{z^{5}}{5!}+\left(1.002561819443906\;\;+0.000100213530804i\right)\frac{z^{6}}{6!}+\left(1.001961004676568+0.000037357996541i\right)\frac{z^{7}}{7!}\;\;+\left(0.998040411547603+0.066628880208108i\right)\frac{z^{8}}{8!}+\left(0.998769798758987+0.058815505018775i\right)\frac{z^{9}}{9!}.$

**Fig. 2:**
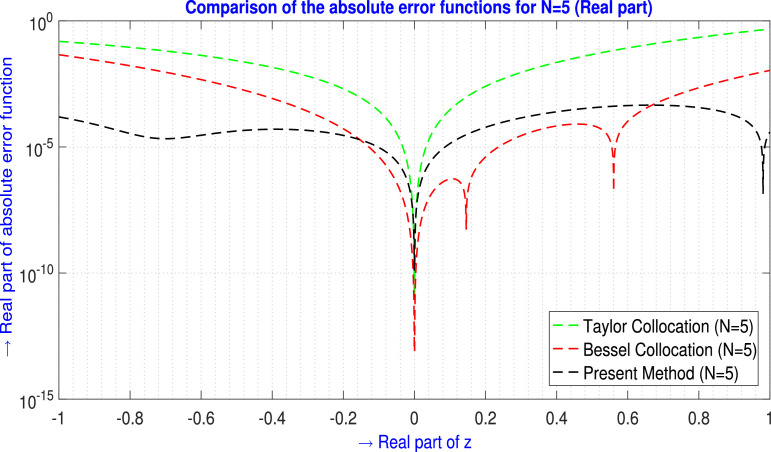
Visual depiction of the absolute error analysis of Example 1 for N=5 (ℜe part).

**Fig. 3:**
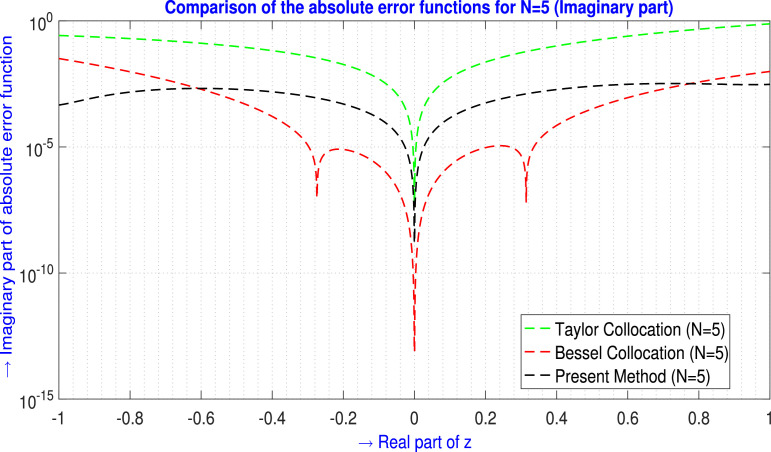
Visual depiction of the absolute error analysis of Example 1 for N=5 (ℑm part).

For N=5,9 the tabular comparison and for N=5 the graphical comparison, the absolute error produced by the present method is compared with the outcomes generated by the Taylor Collocation method [24] and the Bessel Collocation method [25] are shown in Table 1 and in Fig. 2 for ℜe part, and in Table 2 and in Fig. 3 for ℑm part.

Example 2 Let us examine the second-order non-homogeneous CDE that is linear and has variable coefficients [24], [25],

(33) $$f^{{}^{\prime \prime }}\left(z\right)+zf^{{}^{\prime }}\left(z\right)+2zf\left(z\right)=2z\sin z+z\cos z-\sin z;\;z\;\in \;C.$$

Where $m=2,\;Q_{0}\left(z\right)=2z,\;Q_{1}\left(z\right)=z,\;Q_{2}\left(z\right)=1,\;h\left(z\right)=2z\sin z+z\cos z-\sin z$ and subject to the initial conditions are

(34) $$f\left(0\right)=0,\;f^{\prime }\left(0\right)=1.$$

The corresponding transcendental entire solution of Eq. (33) is f(z)=sinz. Assume the Galerkin integral domain $D=\left\{z\;\in \;C,\;z=x+iy,i=\sqrt{-1};\;-1\leq x\leq 1,\;-1\leq y\leq 1\right\}$. For N=5, by applying the proposed method discussed in Method details: Phase 1 and Method details: Phase 2 section, we obtain the approximate solution of the problem (33) is

**Table 1:** Absolute error $E_{N}\left(z\right)$ analysis of Example 1 (ℜe part) for N=5,9.

$z_{j}$	Taylor Collocation [24]$E_{5}\left(z_{j}\right)$(ℜe part)	Bessel Collocation [25]$E_{5}\left(z_{j}\right)$(ℜe part)	Present method $E_{5}\left(z_{j}\right)$(ℜe part)
−1.00−1.00i	$1.5455878\times 10^{-1}$	$4.56461167157\times 10^{-2}$	$1.55825712363\times 10^{-4}$
−0.60−0.60i	$4.306944\times 10^{-2}$	$4.69890942146\times 10^{-3}$	$2.92740621909\times 10^{-5}$
−0.20−0.10i	$7.56259\times 10^{-3}$	$3.53588372881\times 10^{-6}$	$1.70147084216\times 10^{-4}$
−0.20−0.20i	$2.021649\times 10^{-3}$	$5.28547892122\times 10^{-5}$	$2.88995538694\times 10^{-5}$
−0.10−0.20i	$6.3395028\times 10^{-3}$	$1.17953217994\times 10^{-5}$	$2.07165233691\times 10^{-4}$
−0.10+0.20i	$6.3395028\times 10^{-3}$	$1.17953217994\times 10^{-5}$	$1.88735059177\times 10^{-4}$
−0.10−0.10i	$2.685866\times 10^{-4}$	$4.08981352739\times 10^{-6}$	$9.54656886026\times 10^{-6}$
−0.10+0.10i	$2.685866\times 10^{-4}$	$4.08981352739\times 10^{-6}$	$1.52868641185\times 10^{-5}$
0.00+0.00i	0	0	0
0.10+0.10i	$3.00956\times 10^{-4}$	$5.34405858898\times 10^{-7}$	$1.37335135544\times 10^{-5}$
0.10−0.10i	$3.00956\times 10^{-4}$	$5.34405858898\times 10^{-7}$	$8.60026504344\times 10^{-6}$
0.10−0.20i	$9.461532\times 10^{-3}$	$5.89808309592\times 10^{-6}$	$2.18213126268\times 10^{-4}$
0.10+0.20i	$9.461532\times 10^{-3}$	$5.89808309592\times 10^{-6}$	$2.37584226249\times 10^{-4}$
0.20+0.20i	$2.545070\times 10^{-3}$	$4.02982871539\times 10^{-6}$	$6.05804000660\times 10^{-5}$
0.20+0.10i	$8.12515\times 10^{-3}$	$3.01135500091\times 10^{-6}$	$1.81330834974\times 10^{-4}$
0.60+0.60i	$8.494471\times 10^{-2}$	$1.04420502024\times 10^{-4}$	$4.39688872244\times 10^{-4}$
1.00+1.00i	$4.774621\times 10^{-1}$	$1.08853442660\times 10^{-2}$	$3.93946395961\times 10^{-5}$

$L^{\infty }\;norm$→	$4.774621\times 10^{-1}$	$4.56461167157\times 10^{-2}$	$4.39688872244\times 10^{-4}$

$z_{j}$	Taylor Collocation [24]$E_{9}\left(z_{j}\right)$(ℜe part)	Bessel Collocation [25]$E_{9}\left(z_{j}\right)$(ℜe part)	Present method$E_{9}\left(z_{j}\right)$(ℜe part)

−1.00−1.00i	$1.108733182\times 10^{-1}$	$3.76537324165\times 10^{-4}$	$2.57859638130\times 10^{-9}$
−0.60−0.60i	$1.45548425\times 10^{-2}$	$6.06258539787\times 10^{-6}$	$7.58469130873\times 10^{-9}$
−0.20−0.10i	$3.294138\times 10^{-4}$	$2.45158049416\times 10^{-9}$	$2.26350818464\times 10^{-8}$
−0.20−0.20i	$1.061492\times 10^{-4}$	$4.87937790172\times 10^{-9}$	$9.44547400322\times 10^{-10}$
−0.10−0.20i	$3.063314\times 10^{-4}$	$1.05705222352\times 10^{-9}$	$2.49267239421\times 10^{-8}$
−0.10+0.20i	$3.063314\times 10^{-4}$	$1.05705244557\times 10^{-9}$	$3.61462878580\times 10^{-8}$
−0.10−0.10i	$1.9201\times 10^{-6}$	$3.5108893570\times 10^{-10}$	$6.30457370627\times 10^{-10}$
−0.10+0.10i	$1.9201\times 10^{-6}$	$3.5108893570\times 10^{-10}$	$1.51172321082\times 10^{-9}$
0.00+0.00i	0	0	0
0.10+0.10i	$3.429\times 10^{-5}$	$1.4785728197\times 10^{-11}$	$1.29170784506\times 10^{-9}$
0.10−0.10i	$3.429\times 10^{-5}$	$1.4785728197\times 10^{-11}$	$6.39311490701\times 10^{-10}$
0.10−0.20i	$4.95029\times 10^{-4}$	$1.2836420815\times 10^{-10}$	$4.06109024591\times 10^{-8}$
0.10+0.20i	$4.95029\times 10^{-4}$	$1.2836420815\times 10^{-10}$	$2.88975497779\times 10^{-8}$
0.20+0.20i	$4.11766\times 10^{-4}$	$1.7463808177\times 10^{-11}$	$5.50078312939\times 10^{-9}$
0.20+0.10i	$3.58652\times 10^{-4}$	$3.4350300381\times 10^{-11}$	$2.34756393087\times 10^{-8}$
0.60+0.60i	$2.7396252\times 10^{-2}$	$1.5724670343\times 10^{-7}$	$1.08960406759\times 10^{-8}$
1.00+1.00i	$2.12823672\times 10^{-1}$	$1.8848182780\times 10^{-5}$	$7.60426705103\times 10^{-10}$

$L^{\infty }\;norm$→	$2.12823672\times 10^{-1}$	$3.76537324165\times 10^{-4}$	$4.06109024591\times 10^{-8}$

**Table 2:** Absolute error $E_{N}\left(z\right)$ analysis of Example 1 (ℑm part) for N=5,9.

$z_{j}$	Taylor Collocation [24]$E_{5}\left(z_{j}\right)$(ℑm part)	Bessel Collocation [25]$E_{5}\left(z_{j}\right)$(ℑm part)	Present method $E_{5}\left(z_{j}\right)$(ℑm part)
−1.00−1.00i	$2.6405612\times 10^{-1}$	$3.20625005945\times 10^{-2}$	$4.61134911108\times 10^{-4}$
−0.60−0.60i	$1.296049635\times 10^{-1}$	$1.93852368188\times 10^{-3}$	$2.09682236332\times 10^{-3}$
−0.20−0.10i	$9.025824\times 10^{-3}$	$2.46106053944\times 10^{-5}$	$2.82627329290\times 10^{-4}$
−0.20−0.20i	$1.8727366\times 10^{-2}$	$7.97723721643\times 10^{-6}$	$5.05755573585\times 10^{-4}$
−0.10−0.20i	$1.0687015\times 10^{-2}$	$1.50108726619\times 10^{-5}$	$2.62174108542\times 10^{-4}$
−0.10+0.20i	$1.0687015\times 10^{-2}$	$1.50108726619\times 10^{-5}$	$3.13020553920\times 10^{-4}$
−0.10−0.10i	$4.964118\times 10^{-3}$	$1.80962075498\times 10^{-6}$	$1.36401933341\times 10^{-4}$
−0.10+0.10i	$4.964118\times 10^{-3}$	$1.80962075498\times 10^{-6}$	$1.42214405880\times 10^{-4}$
0.00+0.00i	0	0	0
0.10+0.10i	$5.532334\times 10^{-3}$	$1.831842975447\times 10^{-6}$	$1.42946035745\times 10^{-4}$
0.10−0.10i	$5.532334\times 10^{-3}$	$1.831842975447\times 10^{-6}$	$1.46555754649\times 10^{-4}$
0.10−0.20i	$1.011738\times 10^{-2}$	$6.199232248094\times 10^{-6}$	$3.00371756593\times 10^{-4}$
0.10+0.20i	$1.011738\times 10^{-2}$	$6.199232248094\times 10^{-6}$	$2.63978549581\times 10^{-4}$
0.20+0.20i	$2.325711\times 10^{-2}$	$9.399457632647\times 10^{-6}$	$5.56436485635\times 10^{-4}$
0.20+0.10i	$1.2161465\times 10^{-2}$	$3.156056383443\times 10^{-6}$	$3.10634103809\times 10^{-4}$
0.60+0.60i	$2.4723143\times 10^{-1}$	$9.018303218719\times 10^{-4}$	$3.05961727471\times 10^{-3}$
1.00+1.00i	$7.63417273\times 10^{-1}$	$9.857912120237\times 10^{-3}$	$3.03618060646\times 10^{-3}$

$L^{\infty }\;norm$→	$7.63417273\times 10^{-1}$	$3.20625005945\times 10^{-2}$	$3.05961727471\times 10^{-3}$

$z_{j}$	Taylor Collocation [24]$E_{9}\left(z_{j}\right)$(ℑm part)	Bessel Collocation [25]$E_{9}\left(z_{j}\right)$(ℑm part)	Present method $E_{9}\left(z_{j}\right)$(ℑm part)

−1.00−1.00i	$4.026018\times 10^{-2}$	$9.03074377008\times 10^{-5}$	$1.88617719668\times 10^{-9}$
−0.60−0.60i	$7.679373\times 10^{-3}$	$5.48651788112\times 10^{-6}$	$1.46971560696\times 10^{-8}$
−0.20−0.10i	$4.924492\times 10^{-4}$	$2.79223615062\times 10^{-9}$	$3.99808868758\times 10^{-8}$
−0.20−0.20i	$8.613902\times 10^{-4}$	$7.48862591382\times 10^{-9}$	$6.44273901376\times 10^{-8}$
−0.10−0.20i	$4.202928\times 10^{-4}$	$1.40807795978\times 10^{-9}$	$3.95198737399\times 10^{-8}$
−0.10+0.20i	$4.202928\times 10^{-4}$	$1.40807795978\times 10^{-9}$	$4.78513258255\times 10^{-8}$
−0.10−0.10i	$2.3079652\times 10^{-4}$	$8.6068374649\times 10^{-11}$	$1.98854405035\times 10^{-8}$
−0.10+0.10i	$2.3079652\times 10^{-4}$	$8.6068374649\times 10^{-11}$	$2.25097428782\times 10^{-8}$
0.00+0.00i	0	0	0
0.10+0.10i	$2.656783\times 10^{-4}$	$3.0673394380\times 10^{-11}$	$2.06909033500\times 10^{-8}$
0.10−0.10i	$2.656783\times 10^{-4}$	$3.0673394380\times 10^{-11}$	$2.32382756256\times 10^{-8}$
0.10−0.20i	$3.784386\times 10^{-4}$	$8.5109169711\times 10^{-11}$	$4.63237345777\times 10^{-8}$
0.10+0.20i	$3.784386\times 10^{-4}$	$8.5109169711\times 10^{-11}$	$3.94731838006\times 10^{-8}$
0.20+0.20i	$1.124509\times 10^{-3}$	$1.5459328261\times 10^{-11}$	$7.01385834213\times 10^{-8}$
0.20+0.10i	$6.947382\times 10^{-4}$	$1.4565446071\times 10^{-11}$	$4.38067448607\times 10^{-8}$
0.60+0.60i	$1.0193823\times 10^{-2}$	$6.6743245152\times 10^{-8}$	$6.10843735118\times 10^{-8}$
1.00+1.00i	$9.404928\times 10^{-3}$	$4.4066271108\times 10^{-5}$	$1.21583262896\times 10^{-7}$

$L^{\infty }\;norm$→	$4.026018\times 10^{-2}$	$9.03074377008\times 10^{-5}$	$1.21583262896\times 10^{-7}$

$\tilde{f_{5}}\left(z\right)=z+\left(-0.000079478911350-0.000166927143302i\right)\frac{z^{2}}{2!}+\left(-1.007913167448577+0.001537720882906i\right)\frac{z^{3}}{3!}+\left(0.002007654905662+0.001231229067032i\right)\frac{z^{4}}{4!}+\left(0.992600221137810-0.113878146670256i\right)\frac{z^{5}}{5!}.$

Similarly, we can also calculate the approximate solution of Eq. (33), we have

$\tilde{f_{9}}\left(z\right)=z+\left(-0.000000001658373+0.000000000384629i\right)\frac{z^{2}}{2!}+\left(-0.999998866004469-0.000000123584896i\right)\frac{z^{3}}{3!}+\left(-0.000000015631961-0.000000136781017i\right)\frac{z^{4}}{4!}+\left(1.000003367852359+0.000050213858219i\right)\frac{z^{5}}{5!}\;\;+\left(0.000000733950363+0.000000596287442i\right)\frac{z^{6}}{6!}+\left(-1.001957705979327+0.000099883639024i\right)\frac{z^{7}}{7!}+\left(-0.000001758698847+0.000002970752088i\right)\frac{z^{8}}{8!}+\left(0.998048397547541-0.058790820037819i\right)\frac{z^{9}}{9!}.$

**Fig. 4:**
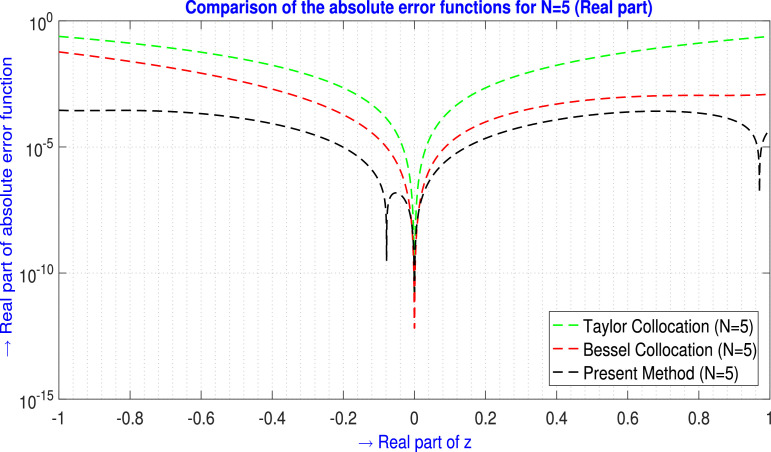
Visual depiction of the absolute error analysis of Example 2 for N=5 (ℜe part).

**Fig. 5:**
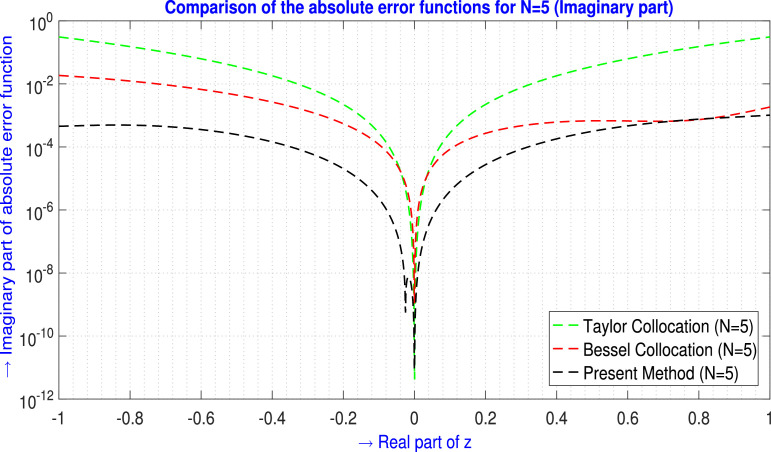
Visual depiction of the absolute error analysis of Example 2 for N=5 (ℑm part).

For N=5,9 the tabular comparison and for N=5 the graphical comparison, the absolute error produced by the present method is compared with the outcomes generated by the Taylor Collocation method [24] and the Bessel Collocation method [25] are shown in Table 3 and in Fig. 4 for ℜe part, and in Table 4 and in Fig. 5 for ℑm part. From the above discussion, the tables and the figures claim more approximation accuracy of the proposed method than the mentioned methods.

Example 3 Let us examine the fourth-order non-homogeneous CDE that is linear and has variable coefficients [25],

(35) $$f^{{}^{\prime \prime \prime \prime }}\left(z\right)-2zf^{{}^{\prime \prime }}\left(z\right)+zf\left(z\right)=24+19z+2z^{2}-29z^{3}+z^{5}\;z\;\in \;C.$$

Where $m=4,\;Q_{0}\left(z\right)=z,\;Q_{1}\left(z\right)=0,\;Q_{2}\left(z\right)=-2z,\;Q_{3}\left(z\right)=0,\;Q_{4}\left(z\right)=1,\;h\left(z\right)=24+19z+2z^{2}-29z^{3}+z^{5}$ and subject to the conditions are

(36) $$f\left(0\right)=-1,\;f^{\prime }\left(0\right)=2,\;f\left(1\right)=-3,\;f^{\prime }\left(1\right)=-4.$$

The corresponding exact solution is a fourth-degree polynomial that is $f\left(z\right)=-1+2z-5z^{2}+z^{4}$.

Assume the Galerkin integral domain $D=\left\{z\;\in \;C,\;z=x+iy,i=\sqrt{-1};\;-1\leq x\leq 1,\;-1\leq y\leq 1\right\}$.

**Table 3:** Absolute error $E_{N}\left(z\right)$ analysis of Example 2 (ℜe part) for N=5,9.

$z_{j}$	Taylor Collocation [24]$E_{5}\left(z_{j}\right)$(ℜe part)	Bessel Collocation [25]$E_{5}\left(z_{j}\right)$(ℜe part)	Present method $E_{5}\left(z_{j}\right)$(ℜe part)
−1.00−1.00i	$2.4326713\times 10^{-1}$	$5.89986041904\times 10^{-2}$	$2.85970519951\times 10^{-4}$
−0.60−0.60i	$5.740202\times 10^{-2}$	$8.45000949478\times 10^{-3}$	$2.10298845644\times 10^{-4}$
−0.20−0.10i	$2.81797\times 10^{-4}$	$1.64846642128\times 10^{-4}$	$7.01455471542\times 10^{-6}$
−0.20−0.20i	$2.212166\times 10^{-3}$	$1.90988842115\times 10^{-4}$	$9.74400536351\times 10^{-6}$
−0.10−0.20i	$1.532345\times 10^{-3}$	$2.54330433371\times 10^{-4}$	$1.00394364354\times 10^{-5}$
−0.10+0.20i	$1.532345\times 10^{-3}$	$2.54330433371\times 10^{-4}$	$1.66588489879\times 10^{-5}$
−0.10−0.10i	$2.775133\times 10^{-4}$	$2.06556950317\times 10^{-5}$	$4.54004234077\times 10^{-7}$
−0.10+0.10i	$2.775133\times 10^{-4}$	$2.06556950319\times 10^{-5}$	$4.89361311986\times 10^{-6}$
0.00+0.00i	0	$8.5541519885\times 10^{-13}$	0
0.10+0.10i	$2.775107\times 10^{-4}$	$1.4879698500\times 10^{-5}$	$3.72562526995\times 10^{-6}$
0.10−0.10i	$2.775107\times 10^{-4}$	$1.4879698500\times 10^{-5}$	$1.48814842361\times 10^{-6}$
0.10−0.20i	$1.530695\times 10^{-3}$	$6.2325482521\times 10^{-5}$	$1.20027715801\times 10^{-5}$
0.10+0.20i	$1.530695\times 10^{-3}$	$6.2325482521\times 10^{-5}$	$1.92300221186\times 10^{-5}$
0.20+0.20i	$2.212125\times 10^{-3}$	$9.8572923268\times 10^{-5}$	$2.20274275447\times 10^{-5}$
0.20+0.10i	$2.80156\times 10^{-4}$	$1.3159328847\times 10^{-4}$	$3.08519534006\times 10^{-6}$
0.60+0.60i	$5.739872\times 10^{-2}$	$9.6432020506\times 10^{-4}$	$2.43755696897\times 10^{-4}$
1.00+1.00i	$2.432416\times 10^{-1}$	$1.2386559787\times 10^{-3}$	$4.93934953302\times 10^{-5}$

$L^{\infty }\;norm$→	$2.4326713\times 10^{-1}$	$5.89986041904\times 10^{-2}$	$2.85970519951\times 10^{-4}$

$z_{j}$	Taylor Collocation [24]$E_{9}\left(z_{j}\right)$(ℜe part)	Bessel Collocation [25]$E_{9}\left(z_{j}\right)$(ℜe part)	Present method $E_{9}\left(z_{j}\right)$(ℜe part)

−1.00−1.00i	$1.2078499\times 10^{-2}$	$4.115916475067\times 10^{-5}$	$1.74967622014\times 10^{-8}$
−0.60−0.60i	$2.484785\times 10^{-3}$	$9.820587112296\times 10^{-6}$	$1.20111606850\times 10^{-8}$
−0.20−0.10i	$1.1912\times 10^{-5}$	$4.429130137650\times 10^{-8}$	$4.51597524796\times 10^{-10}$
−0.20−0.20i	$8.97123\times 10^{-5}$	$1.804838386798\times 10^{-7}$	$2.22042272375\times 10^{-9}$
−0.10−0.20i	$6.22013\times 10^{-5}$	$1.093696364723\times 10^{-7}$	$1.95549871291\times 10^{-9}$
−0.10+0.20i	$6.22013\times 10^{-5}$	$1.093696364723\times 10^{-7}$	$2.23261008978\times 10^{-9}$
−0.10−0.10i	$1.11861\times 10^{-5}$	$1.616685911531\times 10^{-8}$	$3.17901463180\times 10^{-10}$
−0.10+0.10i	$1.11861\times 10^{-5}$	$1.616685925409\times 10^{-8}$	$4.41483525557\times 10^{-10}$
0.00+0.00i	0	$6.49920795016\times 10^{-13}$	0
0.10+0.10i	$1.11835\times 10^{-5}$	$7.01703836702\times 10^{-9}$	$3.25059744464\times 10^{-10}$
0.10−0.10i	$1.11835\times 10^{-5}$	$7.01703836702\times 10^{-9}$	$4.33283141427\times 10^{-10}$
0.10−0.20i	$6.05515\times 10^{-5}$	$2.45374184859\times 10^{-9}$	$2.13889327312\times 10^{-9}$
0.10+0.20i	$6.05515\times 10^{-5}$	$2.45374230656\times 10^{-9}$	$1.94741138691\times 10^{-9}$
0.20+0.20i	$8.96715\times 10^{-5}$	$3.44256884110\times 10^{-8}$	$2.24201153179\times 10^{-9}$
0.20+0.10i	$1.0271\times 10^{-5}$	$3.54173038119\times 10^{-8}$	$4.14418753795\times 10^{-10}$
0.60+0.60i	$2.481483\times 10^{-3}$	$1.98254734962\times 10^{-7}$	$1.10180060964\times 10^{-8}$
1.00+1.00i	$1.2053017\times 10^{-2}$	$4.60677713531\times 10^{-7}$	$1.20032742658\times 10^{-9}$

$L^{\infty }\;norm$→	$1.2078499\times 10^{-2}$	$4.115916475067\times 10^{-5}$	$1.74967622014\times 10^{-8}$

**Table 4:** Absolute error $E_{N}\left(z\right)$ analysis of Example 2 (ℑm part) for N=5,9.

$z_{j}$	Taylor Collocation [24]$E_{5}\left(z_{j}\right)$(ℑm part)	Bessel Collocation [25]$E_{5}\left(z_{j}\right)$(ℑm part)	Present method $E_{5}\left(z_{j}\right)$(ℑm part)
−1.00−1.00i	$3.09312\times 10^{-1}$	$1.85893527304\times 10^{-2}$	$4.53596288293\times 10^{-4}$
−0.60−0.60i	$6.25585\times 10^{-2}$	$6.68868421674\times 10^{-3}$	$3.55780327721\times 10^{-4}$
−0.20−0.10i	$1.52585\times 10^{-3}$	$3.19933894691\times 10^{-4}$	$9.73150983529\times 10^{-6}$
−0.20−0.20i	$2.23548\times 10^{-3}$	$5.46524065895\times 10^{-4}$	$2.04215934919\times 10^{-5}$
−0.10−0.20i	$2.73608\times 10^{-4}$	$1.68336224113\times 10^{-4}$	$1.21921823707\times 10^{-6}$
−0.10+0.20i	$2.73608\times 10^{-4}$	$1.68336224113\times 10^{-4}$	$1.01332516060\times 10^{-5}$
−0.10−0.10i	$2.78721\times 10^{-4}$	$1.19731980447\times 10^{-4}$	$2.29471931160\times 10^{-6}$
−0.10+0.10i	$2.78721\times 10^{-4}$	$1.19731980447\times 10^{-4}$	$1.38653178968\times 10^{-6}$
0.00+0.00i	0	$2.21437262029\times 10^{-28}$	0
0.10+0.10i	$2.77624\times 10^{-4}$	$8.46333017160\times 10^{-5}$	$3.92533850752\times 10^{-6}$
0.10−0.10i	$2.77624\times 10^{-4}$	$8.46333017160\times 10^{-5}$	$2.93506904779\times 10^{-6}$
0.10−0.20i	$2.75787\times 10^{-4}$	$2.05738371287\times 10^{-4}$	$1.61657156734\times 10^{-6}$
0.10+0.20i	$2.75787\times 10^{-4}$	$2.05738371287\times 10^{-4}$	$1.36086931283\times 10^{-7}$
0.20+0.20i	$2.2311\times 10^{-3}$	$2.70937062759\times 10^{-4}$	$2.74365619024\times 10^{-5}$
0.20+0.10i	$1.52364\times 10^{-3}$	$1.23452638561\times 10^{-4}$	$1.75887713028\times 10^{-5}$
0.60+0.60i	$6.2519\times 10^{-2}$	$6.68465941159\times 10^{-4}$	$4.66194239589\times 10^{-4}$
1.00+1.00i	$3.09202\times 10^{-2}$	$1.84717548590\times 10^{-3}$	$1.02296380001\times 10^{-3}$

$L^{\infty }\;norm$→	$3.09312\times 10^{-1}$	$1.85893527304\times 10^{-2}$	$1.02296380001\times 10^{-3}$

$z_{j}$	Taylor Collocation [24]$E_{9}\left(z_{j}\right)$(ℑm part)	Bessel Collocation [25]$E_{9}\left(z_{j}\right)$(ℑm part)	Present method $E_{9}\left(z_{j}\right)$(ℑm part)

−1.00−1.00i	$1.03425\times 10^{-2}$	$2.48152172653\times 10^{-4}$	$1.85987290531\times 10^{-8}$
−0.60−0.60i	$2.36461\times 10^{-3}$	$9.64545246917\times 10^{-6}$	$1.70240876063\times 10^{-8}$
−0.20−0.10i	$6.26632\times 10^{-5}$	$1.61341194057\times 10^{-7}$	$1.91601385659\times 10^{-9}$
−0.20−0.20i	$9.13166\times 10^{-5}$	$1.90064818800\times 10^{-7}$	$2.85283863781\times 10^{-9}$
−0.10−0.20i	$1.0171\times 10^{-5}$	$2.27097510518\times 10^{-8}$	$3.21724716929\times 10^{-11}$
−0.10+0.20i	$1.0171\times 10^{-5}$	$2.27097510518\times 10^{-8}$	$7.67970593828\times 10^{-10}$
−0.10−0.10i	$1.17155\times 10^{-5}$	$4.33176848557\times 10^{-8}$	$4.15969033987\times 10^{-10}$
−0.10+0.10i	$1.17155\times 10^{-5}$	$4.33176844949\times 10^{-8}$	$3.71587874864\times 10^{-10}$
0.00+0.00i	0	$4.44685976728\times 10^{-27}$	0
0.10+0.10i	$1.06186\times 10^{-5}$	$2.27537023805\times 10^{-8}$	$3.87344639278\times 10^{-10}$
0.10−0.10i	$1.06186\times 10^{-5}$	$2.27537023805\times 10^{-8}$	$3.33844698485\times 10^{-10}$
0.10−0.20i	$1.23496\times 10^{-5}$	$5.64710605222\times 10^{-8}$	$8.27722774918\times 10^{-10}$
0.10+0.20i	$1.23496\times 10^{-5}$	$5.64710598283\times 10^{-8}$	$3.43136409355\times 10^{-11}$
0.20+0.20i	$8.6929\times 10^{-5}$	$5.89471798040\times 10^{-8}$	$2.79208080708\times 10^{-9}$
0.20+0.10i	$6.04542\times 10^{-5}$	$2.75819321549\times 10^{-8}$	$1.86596570940\times 10^{-9}$
0.60+0.60i	$2.31512\times 10^{-3}$	$1.56472432677\times 10^{-7}$	$2.10176396896\times 10^{-8}$
1.00+1.00i	$1.02328\times 10^{-2}$	$2.25562201583\times 10^{-7}$	$4.69233857440\times 10^{-8}$

$L^{\infty }\;norm$→	$1.03425\times 10^{-2}$	$2.48152172653\times 10^{-4}$	$4.69233857440\times 10^{-8}$

For N=4, by applying the proposed method discussed in Method details: Phase 1 and Method details: Phase 2 section, we obtain the required augmented matrix as follows:

$$\left[K^{*}:C^{*}\right]=\left[\begin{array}{ccccccc} \;0 & \frac{-4}{3}+\frac{4i}{3} & 0 & \frac{12}{5}-\frac{44i}{15} & 2+2i & : & \frac{163}{3}+\frac{152i}{3}\;\;\\ \;1 & 0 & 0 & 0 & 0 & : & -1.00\;\;\\ \;0 & 1 & 0 & 0 & 0 & : & 2.00\;\;\\ \;1 & 1 & \frac{1}{2} & \frac{1}{6} & \frac{1}{24} & : & -3.00\;\;\\ \;0 & 1 & 1 & \frac{1}{2} & \frac{1}{6} & : & -4.00\; \end{array}\right].$$

By solving the above matrix for unknown coefficients, then the coefficients $a_{j}$ become

$a_{0}=-1.00,\;a_{1}=2.00,\;a_{2}=-10.00,\;a_{3}=0.00,\;\text{and}\;a_{4}=24.00.$ Therefore, the approximate solution of the Eq. (35) becomes

$${\tilde{f}}_{4}\left(z\right)=-1.00+2.00z-5.00z^{2}+z^{4}=f\left(z\right).$$

Example 3 demonstrates how the approximate outcomes generated by the proposed method match perfectly the exact solution of the CDE if the exact solution of the CDE is in the N degree or less than N degree polynomial form.

Example 4 Let us examine the second-order non-linear non-homogeneous CDE [18],

(37) $$f^{{}^{\prime \prime }}+\left(e^{z}-e^{-z}\right){\left(f^{{}^{\prime }}\right)}^{2}-\left(e^{z}+1\right)f=1;\;z\in C$$

where subject to the initial conditions are

(38) $$f\left(0\right)=-1/2,\;f^{\prime }\left(0\right)=1/4.$$

Corresponding exact transcendental entire solution is $f\left(z\right)=-1/\left(e^{z}+1\right)$.

Now consider an approximate solution ${\tilde{f}}_{3}\left(z\right)$ by the N=3 degree Taylor polynomial at $z_{0}=0$ in the following form

(39) $${\tilde{f}}_{3}\left(z\right)=\sum_{j=0}^{3}a_{j}\frac{z^{j}}{j!}.$$

Thus, we have $\;\theta_{j}\left(z\right)=\frac{z^{j}}{j!}\;\text{for}\;j=0,1,2,3$ and $\;\theta_{i}\left(z\right)=\frac{z^{i}}{i!}\;\text{for}\;i=0,1,2,3$.

Assume the Galerkin integral domain, $D=\left\{z\;\in \;C,\;z=x+iy,i=\sqrt{-1};\;-1\leq x\leq 1,\;-1\leq y\leq 1\right\}.$

Since the problem (37) is a non-linear CDE, from Eq. (11) we obtain the corresponding system of a nonlinear equation in the matrix equation as

(40) $$\left[K_{1}\right]\left\{A_{1}\right\}+\left[K_{2}\right]\left\{A_{2}\right\}=\left\{C\right\}$$

where

$K_{1}=\left[\begin{array}{cccc} -3.2699-4.5969i & 1.5803-1.0483i & 3.6833+1.1117i & 0.1984+0.3250i\\ 1.5803-1.0483i & 3.3666-1.7765i & 0.5953+0.9751i & -1.0138+2.0311i\\ 1.6833-0.8883i & 0.5953+0.9751i & -0.1873+1.7134i & -0.2357+0.1372i\\ 0.1984+0.3250i & 0.3196+0.6978i & -0.2357+0.1372i & -0.4359-0.1954i \end{array}\right]$

$A_{1}=\left[a_{0}\;a_{1}\;a_{2}\;a_{3}\right]{}^{T}$

$K_{2}=\left[\begin{array}{cccccc} -6.3211+4.1934i & 0.0000+0.0000i & -2.3812-3.9004i & 0.0000+0.0000i & 0.0000+0.0000i & 0.0000+0.0000i\\ 0.0000+0.0000i & -2.3812-3.9004i & 0.0000+0.0000i & -3.1605+2.0967i & -2.3812-3.9004i & 1.4140-0.8233i\\ -2.3812-3.9004i & 0.0000+0.0000i & 2.8279-1.6466i & 0.0000+0.0000i & 0.0000+0.0000i & 0.0000+0.0000i\\ 0.0000+0.0000i & 0.9426-0.5489i & 0.0000+0.0000i & -0.3969-0.6501i & 0.9426-0.5489i & 0.2089+0.3700i \end{array}\right]$

$A_{2}={\left[a_{1}a_{2}\;a_{1}a_{3}\;a_{2}a_{3}\;a_{1}^{2}\;a_{2}^{2}\;a_{3}^{2}\right]}^{T}$

and

$C={\left[2.0000+2.0000i\;0.0000+0.0000i\;-0.6667+0.6667i\;0.0000+0.0000i\right]}^{T}.$

By applying the initial condition (38) on the Eq. (40) with the help of Eq. (16), we obtain

(41) $$\left[K_{1}^{*}\right]\left\{A_{1}\right\}+\left[K_{2}^{*}\right]\left\{A_{2}\right\}=\left\{C^{*}\right\}$$

where

$K_{1}^{*}=\left[\begin{array}{cccc} -3.2699-4.5969i & 1.5803-1.0483i & 3.6833+1.1117i & 0.1984+0.3250i\\ 1.5803-1.0483i & 3.3666-1.7765i & 0.5953+0.9751i & -1.0138+2.0311i\\ 1.0000+0.0000i & 0.0000+0.0000i & 0.0000+0.0000i & 0.0000+0.0000i\\ 0.0000+0.0000i & 1.0000+0.0000i & 0.0000+0.0000i & 0.0000+0.0000i \end{array}\right]$

$K_{2}^{*}=\left[\begin{array}{cccccc} -6.3211+4.1934i & 0.0000+0.0000i & -2.3812-3.9004i & 0.0000+0.0000i & 0.0000+0.0000i & 0.0000+0.0000i\\ 0.0000+0.0000i & -2.3812-3.9004i & 0.0000+0.0000i & -3.1605+2.0967i & -2.3812-3.9004i & 1.4140-0.8233i\\ 0.0000+0.0000i & 0.0000+0.0000i & 0.0000+0.0000i & 0.0000+0.0000i & 0.0000+0.0000i & 0.0000+0.0000i\\ 0.0000+0.0000i & 0.0000+0.0000i & 0.0000+0.0000i & 0.0000+0.0000i & 0.0000+0.0000i & 0.0000+0.0000i \end{array}\right]$

and

$C^{*}={\left[2.0000+2.0000i\;0.0000+0.0000i\;-0.500+0.0000i\;0.2500+0.0000i\right]}^{T}.$

By solving the system of nonlinear Eq. (41), the unknown Taylor coefficients $a_{j}$ become $a_{0}=-0.500-1.722344475\times 10^{-16}i.\;a_{1}=0.2500+8.978526104\times 10^{-16}i,a_{2}=0.4501617132+0.2833277909i,\;\;\text{and}\;a_{3}=0.7341905712-0.2539949629i.$

Therefore, the approximate solution (39) of Eq. (37) is $\tilde{f_{3}}\left(z\right)=-0.500-1.722344475\times 10^{-16}i+\left(0.2500+8.978526104\times 10^{-16}i\right)z+\left(0.4501617132+0.2833277909i\right)\frac{z^{2}}{2!}+\left(0.7341905712-0.2539949629i\right)\frac{z^{3}}{3!}.$

Similarly, we can also calculate the approximation solution of Eq. (37) for N=4,5. These are

$\tilde{f_{4}}\left(z\right)=-0.5-6.498084614\times 10^{-17}i+\left(0.25+8.521059279\times 10^{-17}i\right)z+\left(0.1663309469-0.6308606503i\right)\frac{z^{2}}{2!}+\left(1.309019477-0.2955715993i\right)\frac{z^{3}}{3!}+\left(3.413537203+0.8541716662i\right)\frac{z^{4}}{4!}$

and

$\tilde{f_{5}}\left(z\right)=-0.5-1.181916589\times 10^{-18}i+\left(0.25-1.775067654\times 10^{-19}i\right)z+\left(-0.000003305144873-0.000009181228736i\right)\frac{z^{2}}{2!}+\left(-0.1320722565+0.0034501747i\right)\frac{z^{3}}{3!}+\left(-0.0003426589705-0.0008109565422i\right)\frac{z^{4}}{4!}+\left(0.2109109257-0.1062329329i\right)\frac{z^{5}}{5!}.$

**Fig. 6:**
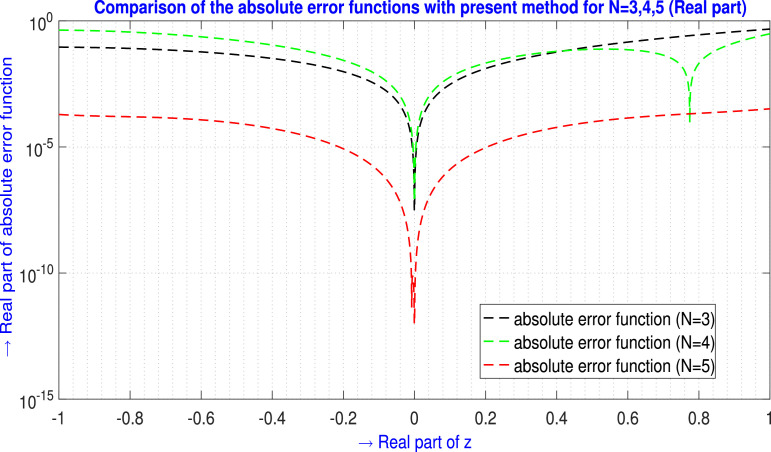
Visual depiction of the absolute error functions with the present method of Example 4 for N=3,4,5 (ℜe part).

**Fig. 7:**
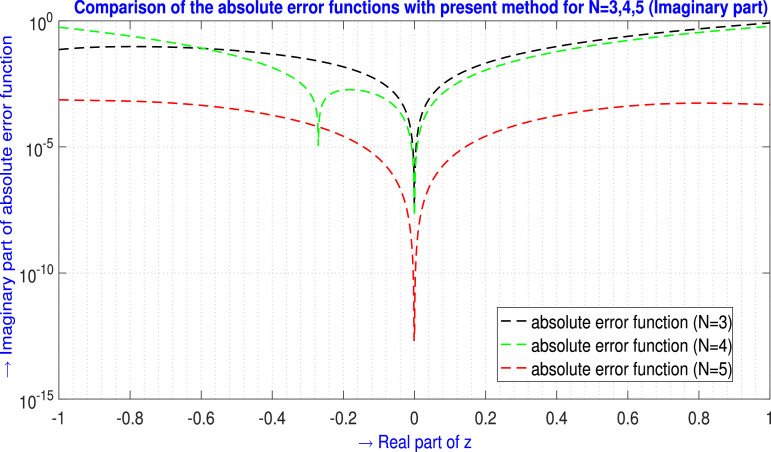
Visual depiction of the absolute error functions with the present method of Example 4 for N=3,4,5 (ℑm part).

For N=3,4,5 the absolute error $E_{N}\left(z\right)$ generated by the present method are shown in Table 5 and in Fig. 6 for ℜe part, and in Table 6 and in Fig. 7 for ℑm part.

Example 5 Let us examine the second-order non-linear non-homogeneous CDE [16],

(42) $$f^{3}f^{{}^{\prime }}+{\left(f^{{}^{\prime }}\right)}^{3}+ff^{{}^{\prime \prime }}+3f^{2}{\left(f^{{}^{\prime \prime }}\right)}^{2}=64e^{4z}+8e^{3z}+4e^{2z};\;z\;\in \;C$$

where subject to the initial conditions are

(43) $$f\left(0\right)=2,\;f^{\prime }\left(0\right)=2.$$

**Table 5:** Absolute error $E_{N}\left(z\right)$ analysis of Example 4 (ℜe part) for N=3,4,5.

$z_{j}$	Absolute error $E_{N}\left(z\right)$ analysis (ℜe part)		
	$E_{3}\left(z_{j}\right)\;\;(ℜe$ part)	$E_{4}\left(z_{j}\right)\;\;(ℜe$ part)	$E_{5}\left(z_{j}\right)\;\;(ℜe$ part)
-1.00-1.00i	9.12210175$\times 10^{-2}$	4.31795313$\times 10^{-1}$	1.94703639$\times 10^{-4}$
-0.90-0.90i	8.80103073$\times 10^{-2}$	4.08792338$\times 10^{-1}$	1.70246213$\times 10^{-4}$
-0.80-0.80i	8.10764674$\times 10^{-2}$	3.61981716$\times 10^{-1}$	1.58242201$\times 10^{-4}$
-0.70-0.70i	7.11610504$\times 10^{-2}$	3.01161415$\times 10^{-1}$	1.43632273$\times 10^{-4}$
-0.60-0.60i	5.91154570$\times 10^{-2}$	2.34654142$\times 10^{-1}$	1.19582451$\times 10^{-4}$
-0.50-0.50i	4.58883571$\times 10^{-2}$	1.69319919$\times 10^{-1}$	8.75903101$\times 10^{-5}$
-0.40-0.40i	3.25096110$\times 10^{-2}$	1.10572163$\times 10^{-1}$	5.40924242$\times 10^{-5}$
-0.30-0.30i	2.00733527$\times 10^{-2}$	6.23946022$\times 10^{-2}$	2.62331769$\times 10^{-5}$
-0.20-0.20i	9.72194476$\times 10^{-3}$	2.73573224$\times 10^{-2}$	8.50546983$\times 10^{-6}$
-0.10-0.10i	2.63162954$\times 10^{-3}$	6.63111334$\times 10^{-3}$	1.08762437$\times 10^{-6}$
0.00+0.00i	0.00000	0.00000	0.00000
0.10+0.10i	3.03492628$\times 10^{-3}$	5.87231509$\times 10^{-3}$	1.28267091$\times 10^{-6}$
0.20+0.20i	1.29442785$\times 10^{-2}$	2.12909765$\times 10^{-2}$	9.42271958$\times 10^{-6}$
0.30+0.30i	3.09256497$\times 10^{-2}$	4.19437644$\times 10^{-2}$	2.88109773$\times 10^{-5}$
0.40+0.40i	5.81552821$\times 10^{-2}$	6.21743947$\times 10^{-2}$	5.99544406$\times 10^{-5}$
0.50+0.50i	9.57755383$\times 10^{-2}$	7.49950482$\times 10^{-2}$	9.93196530$\times 10^{-5}$
0.60+0.60i	1.44880552$\times 10^{-1}$	7.21007195$\times 10^{-2}$	1.40995803$\times 10^{-4}$
0.70+0.70i	2.06500185$\times 10^{-1}$	4.38852617$\times 10^{-2}$	1.80054017$\times 10^{-4}$
0.80+0.80i	2.81583105$\times 10^{-1}$	2.05416962$\times 10^{-2}$	2.16778545$\times 10^{-4}$
0.90+0.90i	3.70980714$\times 10^{-1}$	1.33338671$\times 10^{-1}$	2.60059321$\times 10^{-4}$
1.00+1.00i	4.75434564$\times 10^{-1}$	3.07919747$\times 10^{-1}$	3.27285753$\times 10^{-4}$

$L^{\infty }\;norm$→	4.75434564$\times 10^{-1}$	4.31795313$\times 10^{-1}$	3.27285753$\times 10^{-4}$

**Table 6:** Absolute error $E_{N}\left(z\right)$ analysis of Example 4 (ℑm part) for N=3,4,5.

$z_{j}$	Absolute error $E_{N}\left(z\right)$ analysis (ℑm part)		
	$E_{3}\left(z_{j}\right)\;\;(ℑm$ part)	$E_{4}\left(z_{j}\right)\;\;(ℑm$ part)	$E_{5}\left(z_{j}\right)\;\;(ℑm$ part)
-1.00-1.00i	7.27147628$\times 10^{-2}$	5.58946462$\times 10^{-1}$	7.43492113$\times 10^{-4}$
-0.90-0.90i	9.01205576$\times 10^{-2}$	3.82972581$\times 10^{-1}$	6.96983381$\times 10^{-4}$
-0.80-0.80i	9.57821070$\times 10^{-2}$	2.49380915$\times 10^{-1}$	6.54712150$\times 10^{-4}$
-0.70-0.70i	9.20556334$\times 10^{-2}$	1.51678245$\times 10^{-1}$	5.70944575$\times 10^{-4}$
-0.60-0.60i	8.13112453$\times 10^{-2}$	8.36991376$\times 10^{-2}$	4.47654187$\times 10^{-4}$
-0.50-0.50i	6.59110889$\times 10^{-2}$	3.96277885$\times 10^{-2}$	3.08486537$\times 10^{-4}$
-0.40-0.40i	4.81954325$\times 10^{-2}$	1.40119408$\times 10^{-2}$	1.80656303$\times 10^{-4}$
-0.30-0.30i	3.04760098$\times 10^{-2}$	1.76954077$\times 10^{-3}$	8.41035673$\times 10^{-5}$
-0.20-0.20i	1.50353289$\times 10^{-2}$	1.81057099$\times 10^{-3}$	2.66155254$\times 10^{-5}$
-0.10-0.10i	4.13047212$\times 10^{-3}$	1.07245975$\times 10^{-3}$	3.43966990$\times 10^{-6}$
0.00+0.00i	1.72234447$\times 10^{-16}$	6.49808461$\times 10^{-17}$	1.18191659$\times 10^{-18}$
0.10+0.10i	4.87276214$\times 10^{-3}$	2.22568680$\times 10^{-3}$	3.47874091$\times 10^{-6}$
0.20+0.20i	2.09776082$\times 10^{-2}$	1.10403465$\times 10^{-2}$	2.64474268$\times 10^{-5}$
0.30+0.30i	5.05530986$\times 10^{-2}$	2.94028477$\times 10^{-2}$	8.25089107$\times 10^{-5}$
0.40+0.40i	9.58563157$\times 10^{-2}$	5.99489123$\times 10^{-2}$	1.74793787$\times 10^{-4}$
0.50+0.50i	1.59169768$\times 10^{-1}$	1.04998019$\times 10^{-1}$	2.93244181$\times 10^{-4}$
0.60+0.60i	2.42805188$\times 10^{-1}$	1.66557203$\times 10^{-1}$	4.15000569$\times 10^{-4}$
0.70+0.70i	3.49102846$\times 10^{-1}$	2.46320368$\times 10^{-1}$	5.09280062$\times 10^{-4}$
0.80+0.80i	4.80424886$\times 10^{-1}$	3.45661622$\times 10^{-1}$	5.48220135$\times 10^{-4}$
0.90+0.90i	6.39141418$\times 10^{-1}$	4.65621372$\times 10^{-1}$	5.24981520$\times 10^{-4}$
1.00+1.00i	8.27608664$\times 10^{-1}$	6.06884467$\times 10^{-1}$	4.79783556$\times 10^{-4}$

$L^{\infty }\;norm$→	8.27608664$\times 10^{-1}$	6.06884467$\times 10^{-1}$	7.43492113$\times 10^{-4}$

Corresponding exact transcendental entire solution is $f\left(z\right)=2e^{z}$. Assume the Galerkin integral domain, $D=\left\{z\;\in \;C,\;z=x+iy,i=\sqrt{-1};\;-1\leq x\leq 1,\;-1\leq y\leq 1\right\}.$

Since all the left terms of the problem (42) are non-linear, for better approximation we have to apply a higher degree Taylor polynomial (3). By applying the proposed method and working like the previous problem Example 4, we obtain the approximate outcomes for various values of N=3,5,7 as follows,

${\tilde{f}}_{3}\left(z\right)=2.0+1.35958319\times 10^{-16}i+\left(2.0-2.229822044\times 10^{-16}i\right)z+\left(1.296003129+0.4445017332i\right)\frac{z^{2}}{2!}+\left(3.296225873+0.4425104407i\right)\frac{z^{3}}{3!}$

${\tilde{f}}_{5}\left(z\right)=2.0-1.367168896\times 10^{-17}i+\left(2.0-1.468715595\times 10^{-15}i\right)z+\left(2.014398875-0.06346333426i\right)\frac{z^{2}}{2!}+\left(2.164686133-0.01341524374i\right)\frac{z^{3}}{3!}+\left(2.150249405+0.3559799801i\right)\frac{z^{4}}{4!}+\left(1.82687085+0.9253462085i\right)\frac{z^{5}}{5!}$

and

$\tilde{f_{7}}\left(z\right)=2.0-1.921605847\times 10^{-14}i+\left(2.0-1.89404048\times 10^{-13}i\right)z+\left(1.998968732-0.0002443070988i\right)\frac{z^{2}}{2!}+\left(1.998961413-0.004112936056i\right)\frac{z^{3}}{3!}+\left(2.011369606-0.009140450582i\right)\frac{z^{4}}{4!}+\left(2.061221681+0.001813540748i\right)\frac{z^{5}}{5!}+\left(2.058635165+0.1932847585i\right)\frac{z^{6}}{6!}+\left(1.918077705+0.6304125825i\right)\frac{z^{7}}{7!}.$

**Fig. 8:**
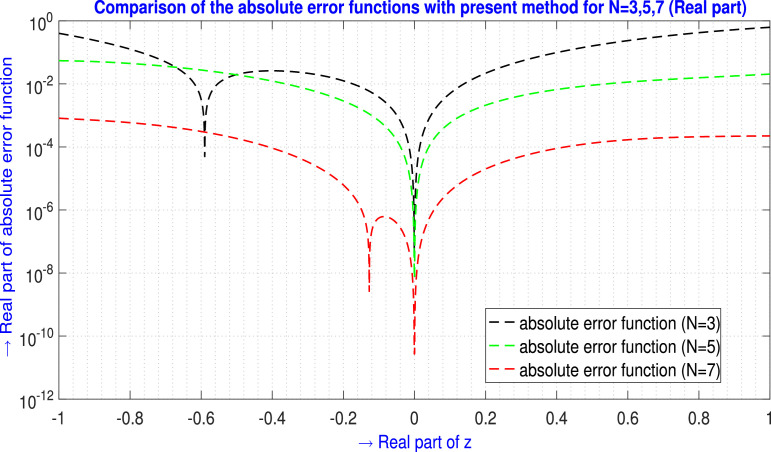
Visual depiction of the absolute error functions with the present method of Example 5 for N=3,5,7 (ℜe part).

**Fig. 9:**
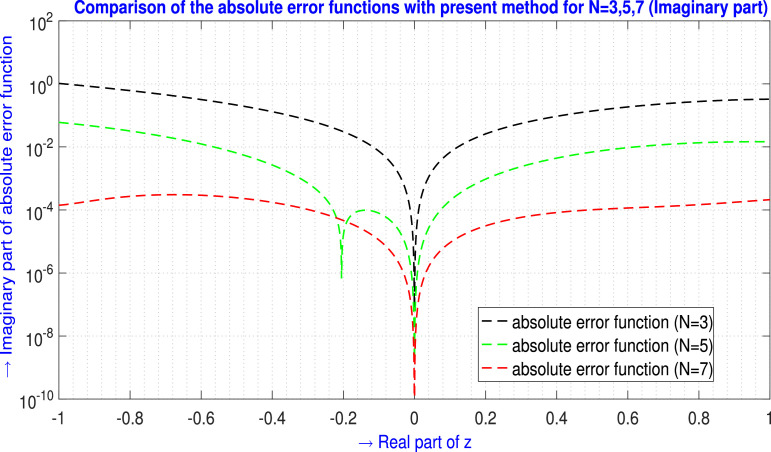
Visual depiction of the absolute error functions with the present method of Example 5 for N=3,5,7 (ℑm part).

Now for N=3,5,7 the absolute error $E_{N}\left(z\right)$ generated by the present method are shown in Table 7 and in Fig. 8 for ℜe part, and in Table 8 and in Fig. 9 for ℑm part.

**Table 7:** Absolute error $E_{N}\left(z\right)$ analysis of Example 5 (ℜe part) for N=3,5,7.

$z_{j}$	Absolute error $E_{N}\left(z\right)$ analysis (ℜe part)		
	$E_{3}\left(z_{j}\right)\;\;(ℜe$ part)	$E_{5}\left(z_{j}\right)\;\;(ℜe$ part)	$E_{7}\left(z_{j}\right)\;\;(ℜe$ part)
-1.00-1.00i	4.04211484$\times 10^{-1}$	5.46973308$\times 10^{-2}$	8.17365239$\times 10^{-4}$
-0.90-0.90i	2.43011014$\times 10^{-1}$	5.13235575$\times 10^{-2}$	7.10864263$\times 10^{-4}$
-0.80-0.80i	1.27495547$\times 10^{-1}$	4.47224516$\times 10^{-2}$	5.91143240$\times 10^{-4}$
-0.70-0.70i	5.00375561$\times 10^{-2}$	3.64450535$\times 10^{-2}$	4.52687224$\times 10^{-4}$
-0.60-0.60i	3.26081238$\times 10^{-3}$	2.77220908$\times 10^{-2}$	3.11176527$\times 10^{-4}$
-0.50-0.50i	1.99062140$\times 10^{-2}$	1.94813171$\times 10^{-2}$	1.86293963$\times 10^{-4}$
-0.40-0.40i	2.61718651$\times 10^{-2}$	1.23699396$\times 10^{-2}$	9.21692603$\times 10^{-5}$
-0.30-0.30i	2.18178852$\times 10^{-2}$	6.78196884$\times 10^{-3}$	3.35881162$\times 10^{-5}$
-0.20-0.20i	1.26314005$\times 10^{-2}$	2.89017748$\times 10^{-3}$	6.23900354$\times 10^{-6}$
-0.10-0.10i	3.83277158$\times 10^{-3}$	6.82186967$\times 10^{-4}$	5.58258273$\times 10^{-7}$
0.00+0.00i	0.000000	0.0000000	0.0000000
0.10+0.10i	4.99059643$\times 10^{-3}$	5.82071389$\times 10^{-4}$	3.95317573$\times 10^{-6}$
0.20+0.20i	2.18620755$\times 10^{-2}$	2.10675218$\times 10^{-3}$	1.99906117$\times 10^{-5}$
0.30+0.30i	5.27925309$\times 10^{-2}$	4.23565379$\times 10^{-3}$	4.98925287$\times 10^{-5}$
0.40+0.40i	9.90030631$\times 10^{-2}$	6.65515886$\times 10^{-3}$	8.98798595$\times 10^{-5}$
0.50+0.50i	1.60684186$\times 10^{-1}$	9.11395386$\times 10^{-3}$	1.32493224$\times 10^{-4}$
0.60+0.60i	2.36928720$\times 10^{-1}$	1.14540761$\times 10^{-2}$	1.69648901$\times 10^{-4}$
0.70+0.70i	3.25674085$\times 10^{-1}$	1.36325560$\times 10^{-2}$	1.95991325$\times 10^{-4}$
0.80+0.80i	4.23657367$\times 10^{-1}$	1.57302960$\times 10^{-2}$	2.11223200$\times 10^{-4}$
0.90+0.90i	5.26386952$\times 10^{-1}$	1.79443689$\times 10^{-2}$	2.19632988$\times 10^{-4}$
1.00+1.00i	6.28135051$\times 10^{-1}$	2.05594357$\times 10^{-2}$	2.24560538$\times 10^{-4}$

$L^{\infty }\;norm$→	6.28135051$\times 10^{-1}$	5.46973308$\times 10^{-2}$	8.17365239$\times 10^{-4}$

**Table 8:** Absolute error $E_{N}\left(z\right)$ analysis of Example 5 (ℑm part) for N=3,5,7.

$z_{j}$	Absolute error $E_{N}\left(z\right)$ analysis (ℑm part)		
	$E_{3}\left(z_{j}\right)\;\;(ℑm$ part)	$E_{5}\left(z_{j}\right)\;\;(ℑm$ part)	$E_{7}\left(z_{j}\right)\;\;(ℑm$ part)
-1.00-1.00i	1.03611560$\times 10^{0}$	6.01045938$\times 10^{-2}$	1.41528255$\times 10^{-4}$
-0.90-0.90i	8.06736405$\times 10^{-1}$	4.54161578$\times 10^{-2}$	2.00690619$\times 10^{-4}$
-0.80-0.80i	6.12934359$\times 10^{-1}$	3.20963001$\times 10^{-2}$	2.70031006$\times 10^{-4}$
-0.70-0.70i	4.51415193$\times 10^{-1}$	2.09823648$\times 10^{-2}$	3.04581051$\times 10^{-4}$
-0.60-0.60i	3.19141665$\times 10^{-1}$	1.24304057$\times 10^{-2}$	2.93049274$\times 10^{-4}$
-0.50-0.50i	2.13331451$\times 10^{-1}$	6.42316081$\times 10^{-3}$	2.43897582$\times 10^{-4}$
-0.40-0.40i	1.31448920$\times 10^{-1}$	2.67192486$\times 10^{-3}$	1.74573442$\times 10^{-4}$
-0.30-0.30i	7.11896499$\times 10^{-2}$	7.11149655$\times 10^{-4}$	1.03758386$\times 10^{-4}$
-0.20-0.20i	3.04564010$\times 10^{-2}$	1.54952933$\times 10^{-5}$	4.63952780$\times 10^{-5}$
-0.10-0.10i	7.32518528$\times 10^{-3}$	7.89612721$\times 10^{-5}$	1.11648744$\times 10^{-5}$
0.00+0.00i	1.35958319$\times 10^{-16}$	1.36716890$\times 10^{-17}$	1.92160585$\times 10^{-14}$
0.10+0.10i	6.75470769$\times 10^{-3}$	1.97194680$\times 10^{-4}$	9.15710610$\times 10^{-6}$
0.20+0.20i	2.58605042$\times 10^{-2}$	9.49403187$\times 10^{-4}$	3.13146474$\times 10^{-5}$
0.30+0.30i	5.54973870$\times 10^{-2}$	2.37420107$\times 10^{-3}$	5.81407275$\times 10^{-5}$
0.40+0.40i	9.36480383$\times 10^{-2}$	4.42390989$\times 10^{-3}$	8.27745943$\times 10^{-5}$
0.50+0.50i	1.37972574$\times 10^{-1}$	6.90075890$\times 10^{-3}$	1.01704221$\times 10^{-4}$
0.60+0.60i	1.85662695$\times 10^{-1}$	9.49264756$\times 10^{-3}$	1.15602408$\times 10^{-4}$
0.70+0.70i	2.33273893$\times 10^{-1}$	1.18308464$\times 10^{-2}$	1.28814242$\times 10^{-4}$
0.80+0.80i	2.76534578$\times 10^{-1}$	1.35707846$\times 10^{-2}$	1.47377576$\times 10^{-4}$
0.90+0.90i	3.10131223$\times 10^{-1}$	1.44968166$\times 10^{-2}$	1.75714220$\times 10^{-4}$
1.00+1.00i	3.27468967$\times 10^{-1}$	1.46515281$\times 10^{-2}$	2.12461960$\times 10^{-4}$

$L^{\infty }\;norm$→	1.03611560$\times 10^{0}$	6.01045938$\times 10^{-2}$	3.04581051$\times 10^{-4}$

## Conclusion

To solve the high-order linear and nonlinear CDEs analytically, is a challenging task. To solve numerically, we provide the TGM in a rectangular domain, and based on the Taylor polynomials. A fascinating feature of the suggested method is its ability to yield precise results in instances when the linear CDE has an exact solution that is represented by a polynomial of degree N or less than N. For the linear CDE, the tabular and graphical comparisons reveal that the method we suggested is more accurate and stable than the existing Collocation method. For the nonlinear CDE, our proposed method goes to the accurate solution when N is sufficiently large enough. Those reveal the validity of our proposed method but it comes with greater computational complexity due to the need to compute higher-order terms.

In the future, we will demonstrate the practical use of the TGM for solving CDEs in real-life problems. For example, the well-known Schrödinger equation in quantum mechanics is a CDE used to describe the behavior of particles at the atomic level.

Present work can also be completed utilizing the Haar Wavelet, Petrov-Galerkin, finite difference, and compact finite difference computations. With a few adjustments, the suggested method can be applied to fractional order CDEs and the system of CDEs with variable coefficients.

## Ethics statements

This article does not contain any studies with human or animal participants.

## CRediT author statement

The author reviewed the results and approved the final version of the manuscript.

## Funding

This research received no external funding.

## Declaration of competing interest

The author declares that they have no known competing financial interests or personal relationships that could have appeared to influence the work reported in this paper.

## Data Availability

Data will be made available on request.
